# Laboratory Testing Implications of Risk-Stratification and Management of COVID-19 Patients

**DOI:** 10.3389/fmed.2021.699706

**Published:** 2021-08-13

**Authors:** Caidong Liu, Ziyu Wang, Wei Wu, Changgang Xiang, Lingxiang Wu, Jie Li, Weiye Hou, Huiling Sun, Youli Wang, Zhenling Nie, Yingdong Gao, Ruisheng Zhang, Haixia Tang, Qianghu Wang, Kening Li, Xinyi Xia, Pengping Li, Shukui Wang

**Affiliations:** ^1^Department of Laboratory Medicine, Nanjing First Hospital, Nanjing Medical University, Nanjing, China; ^2^Department of Bioinformatics, Nanjing Medical University, Nanjing, China; ^3^Department of Laboratory Medicine, First People's Hospital of Jiangxia District of Wuhan, Wuhan, China; ^4^General Clinical Research Center, Nanjing First Hospital, Nanjing Medical University, Nanjing, China; ^5^Department of Critical Care Medicine, Luan Hospital of Chinese Medicine, Lu'an, China; ^6^Jiangsu Key Lab of Cancer Biomarkers, Prevention and Treatment, Collaborative Innovation Center for Personalized Cancer Medicine, Nanjing Medical University, Nanjing, China; ^7^Collaborative Innovation Center for Cardiovascular Disease Translational Medicine, Nanjing, China; ^8^COVID-19 Research Center, Institute of Laboratory Medicine, Jinling Hospital, Nanjing University School of Medicine, Nanjing, China; ^9^Department of Laboratory Medicine and Blood Transfusion, Wuhan Huoshenshan Hospital, Wuhan, China; ^10^Joint Expert Group for COVID-19, Wuhan Huoshenshan Hospital, Wuhan, China

**Keywords:** COVID-19, laboratory testing, diagnosis, monitoring, prediction model

## Abstract

**Objective:** To distinguish COVID-19 patients and non-COVID-19 viral pneumonia patients and classify COVID-19 patients into low-risk and high-risk at admission by laboratory indicators.

**Materials and methods:** In this retrospective cohort, a total of 3,563 COVID-19 patients and 118 non-COVID-19 pneumonia patients were included. There are two cohorts of COVID-19 patients, including 548 patients in the training dataset, and 3,015 patients in the testing dataset. Laboratory indicators were measured during hospitalization for all patients. Based on laboratory indicators, we used the support vector machine and joint random sampling to risk stratification for COVID-19 patients at admission. Based on laboratory indicators detected within the 1st week after admission, we used logistic regression and joint random sampling to develop the survival mode. The laboratory indicators of COVID-10 and non-COVID-19 were also compared.

**Results:** We first identified the significant laboratory indicators related to the severity of COVID-19 in the training dataset. Neutrophils percentage, lymphocytes percentage, creatinine, and blood urea nitrogen with AUC >0.7 were included in the model. These indicators were further used to build a support vector machine model to classify patients into low-risk and high-risk at admission in the testing dataset. Results showed that this model could stratify the patients in the testing dataset effectively (AUC = 0.89). Our model still has good performance at different times (Mean AUC: 0.71, 0.72, 0.72, respectively for 3, 5, and 7 days after admission). Moreover, laboratory indicators detected within the 1st week after admission were able to estimate the probability of death (AUC = 0.95). We identified six indicators with permutation *p* < 0.05, including eosinophil percentage (*p* = 0.007), white blood cell count (*p* = 0.045), albumin (*p* = 0.041), aspartate transaminase (*p* = 0.043), lactate dehydrogenase (*p* = 0.002), and hemoglobin (*p* = 0.031). We could diagnose COVID-19 and differentiate it from other kinds of viral pneumonia based on these laboratory indicators.

**Conclusions:** Our risk-stratification model based on laboratory indicators could help to diagnose, monitor, and predict severity at an early stage of COVID-19. In addition, laboratory findings could be used to distinguish COVID-19 and non-COVID-19.

## Introduction

Coronavirus disease 2019 (COVID-19) has become a serious worldwide problem. It is caused by a novel coronavirus severe acute respiratory syndrome coronavirus (SARS-CoV-2). As of March 1, 2021, there have been 100,221,840 confirmed cases of COVID-19, including 114,040,659 deaths (https://who.sprinklr.com/). The global outbreak of COVID-19 highlights the importance of early and rapid diagnosis, monitoring, risk assessment, and medical resource management in the prevention and control of epidemics (1).

The death of COVID-19 patients is mainly caused by the progression from mild to critical illness (2). Therefore, there is an urgent need for effective methods to predict prognosis early. At present, nucleic acid detection and antibody detection are the main technical approaches for clinical diagnosis of COVID-19 patients, but both of them are affected by many factors, such as sample location, type, quality, and patient condition as well as sample storage, which causes a certain degree of false positives and false negatives (3). Most importantly, they all failed to help judge whether a patient will progress to severe illness (4–6). CT imaging is also a common method, but it lacks specificity and requires a large number of professional technicians, and thus easily exhausts resources when the epidemic is serious. The latest research shows that, based on artificial intelligence methods, CT can be used to diagnose or stratify COVID-19 quickly. However, the accuracy of using CT alone to predict patient severity is limited (7–9).

Previous studies have reported that in the early published 41 COVID-19 cases, five patients presented with varying degrees of myocardial injury, cardiovascular disease patients are more likely to develop into severe patients after COVID-19 infection, and the risk of death is higher (10). The abnormal of different laboratory indicators can represent damage to different organs. For example, NT-proBNP indicates cardiac dysfunction and Alkaline phosphatase (ALP) indicates liver dysfunction. In addition, other laboratory indicators are highly correlated with the risk of disease progression, such as the lymphocyte, IL-6, etc. (11, 12). These findings suggested that laboratory indicators can be used to predict the severity of COVID-19 pneumonia patients. It is of significant importance to perform risk-stratification and management of epidemic disease, especially in countries with a shortage of medical resources, where using limited resources to a greater extent for more critically ill patients will help improve the utilization of medical resources. It is necessary to perform more rigorous testing and clinical observation for patients who tend to have a more severe reaction.

This study aims to identify the laboratory indicators that could predict severity as early as admission, and build a practical risk-stratification model for screening severe COVID-19 patients, as well as predicting the risk of death. This prognostic model based on laboratory indicators could provide important information for the diagnosis, stratification, and monitoring for COVID-19 patients as early as possible.

## Methods

### Data Collection

From December 1, 2019, to February 13, 2020, a total of 548 cases of confirmed COVID-19 patients were collected from the First People's Hospital of Jiangxia District of Wuhan, including 474 moderate COVID-19 patients and 74 severe COVID-19 patients (FPHJ-548 dataset). Three hundred eighty-five COVID-19 patients who received blood tests at admission were included for the analysis. Eighteen non-COVID-19 viral pneumonia cases were also collected from December 1, 2019, to February 13, 2020, in the First People's Hospital of Jiangxia District of Wuhan. One-hundred patients with non-COVID-19 pneumonia were collected from October 1, 2019, to April 40, 2020, in the Nanjing First Hospital. These 118 non-COVID-19 viral pneumonia cases were designated as the nCVP-118 dataset, including 40 patients with parainfluenza virus, 20 patients with the respiratory syncytial virus, 13 patients with influenza A, 29 patients with influenza B, and 16 patients with adenovirus. One thousand four hundred fifty-two moderate and 1,563 severe COVID-19 patients were collected from Wuhan Huoshenshan Hospital as a validated dataset (HSSH-3015) from February 4, 2020, to April 10, 2020. The diagnosis of COVID-19 in these datasets is based on the “New Coronavirus Pneumonia Diagnosis and Treatment Plan (provisional Sixth Edition)” issued by the National Health and Health Commission. Written informed consent was obtained from each patient.

### SVM Approach for Risk-Stratification Based on Laboratory Indicators at Admission

Using the highest severity during hospitalization of each patient in training dataset (FPHJ-548) as labels, an SVM model was constructed to predict severity at admission based on blood test results. The steps of the SVM risk-stratification method are described as follows: (1) The laboratory indicators with AUC>0.7 were selected. The indicators which had no detection data in the testing dataset (HSSH-3015) were excluded. Finally, four laboratory findings (LYMPH%, NEUT%, CREA, and BUN) were used to develop a risk-stratification model. (2) We normalized the original value of each indicator according to the normal range. A normalized value >1 means they exceeded the maximum normal range. A normalized value of <0 indicates that it is below the minimum normal range. A normalized value range from 0 to 1 indicates that it is within the normal range (Equation 1). (3) We predicted the severity of each patient using the SVM model. The basic principle of this method is to find a fractal hyperplane for the training set in the sample space, which will maximize the separation of categories. We defined the distance from the sample to the hyperplane as the risk-stratification score (RSS) (Equation 2). W represents the coefficient of laboratory indicators trained by SVM. X represents the vector of laboratory indicators. We used 5-fold cross-validation in the training dataset (FPHJ-548) to prove the feasibility of risk stratification based on the four indicators. Due to the emergency of the epidemic, only 200 patients in the training dataset without any missing value on laboratory indicators. Finally, we used 200 patients to develop the risk model. To include more patients in the external validation dataset and validate the stability of our model at different time points, we considered the status of laboratory indicators within 3, 5, and 7 days after admission. The total number of patients without any missing laboratory indicators was respectively 2,036, 2,427, and 2,617 within 3, 5, 7 after admission. To match the number of patients in the training dataset (FPHJ-548), we randomly selected 200 patients without replacement within 3, 5, and 7 days after admission from the testing dataset (HSSH-3015 dataset). We used the same method to evaluate the robustness of our model. The process was repeated 50 times. Patients were grouped based on age and sex to validate the model. The prediction performances of AUC were calculated using the predicted values estimated by the model with the combination of selected features as predictors and the status of progression as an outcome.

(1) $$\begin{array}{l} normalizaed\;value\\ =\;\frac{Detected\;value-min\left(normal\;range\right)}{\max\left(normal\;range\right)-min\left(normal\;range\right)} \end{array}$$

(2) $$\begin{array}{l} RSS=\overset{N}{\underset{i=1}{\sum }}W_{i}*X_{i}+B \end{array}$$

### LR Approach for Survival Outcome Based on Laboratory Indicators Within 1 Week Since Admission

To monitor the risk of deaths of severe COVID-19 patients, we randomly split HSSH-3015 into a leave-in training set and a leave-out test set for data analysis at a ratio of ~50%:50% (using a random number generator). To predict the survival outcome early, we only selected laboratory findings within the 1st week after admission. For the training set, 724 survival samples, and 28 deaths were selected. For the matched leave-out test set, 724 survivors, and 27 dead samples were selected. For the training dataset, we randomly selected 28 survivors. We incorporated group sizes of 28 dead individuals and 28 deaths to develop the model by stepwise LR. This random process was repeated 100 times, leading to 100 different model-building. Indicators that were significant in over 10 out of 100 models were considered as potential risk-related factors. Thirteen indicators were involved for the next modeling, including Albumin/Globulin, DD dimer, leukocyte, monocytes, cystatin C, creatinine, lymphocyte, urea nitrogen, thrombin time, prothrombin time, lactate dehydrogenase, fibrinogen, percentage of neutrophils. Then, we trained the LR model by these indicators in the training dataset and validated it in the leave-out test set. RSS was calculated as above (Equation 2). W represents the coefficient of laboratory indicators trained by LR.

### Distinguishing COVID-19 From Non-COVID-19 Based on Laboratory Indicators

Laboratory findings at admission were used to distinguish COVID-19 from non-COVID-19 patients. Thirteen laboratory findings, shared between the FPHJ-548 and nCVP-118 datasets were included, in which six indicators showed a significant difference between COVID-19 and non-COVID-19 patients selected for further analysis (adjust *p* < 0.05). To eliminate the influence of missing values, we only considered patients with no missing values in these indicators. We finally selected 212 patients from FPHJ-548, 99 patients from nCVP-118, and 2,828 patients from HSSH-3015. For FPHJ-548 and nCVP-118, we clustered based on the maximum distance and performed multidimensional scaling and validated by HSSH-3015 and nCVP-118.

### Statistical Analysis

Continuous and categorical variables were presented as median (IQR) and n (%), respectively. We used the Wilcoxon rank-sum test (for continuous quantitative variables) or Fisher's exact test (for categorical variables) to compare differences between moderate and severe patients where appropriate. In the bilateral test, the index of *p* < 0.05 is considered statistically significant. ROC curves and their correspondent AUC of RSS were calculated by R package pROC. The permutation *p*-value was based on 1,000 iterations. In each iteration, we randomly sampled 20 COVID-19 and 20 non-COVID-19 patients and calculated the proportion that fit the trend. Analysis was carried out using the statistical software R (version: 3.6.0). All figures were plotted by ggplot2 package.

## Results

### Study Design

We collected the clinical data of 3,563 COVID-19 patients and 118 non-COVID-19 viral pneumonia (designated as non-COVID-19) to build and validate the risk-stratification model. Specifically, data of 548 patients from the First People's Hospital of Jiangxia District of Wuhan were used as a training dataset (FPHJ-548); data of 3,015 patients from Wuhan Huoshenshan Hospital were used as a testing dataset (HSSH-3015); data of 18 non-COVID-19 viral pneumonia patients from the First People's Hospital of the Jiangxia District of Wuhan and data of 100 non-COVID-19 viral pneumonia patients from Nanjing First Hospital were used to differentiate COVID-19 from non-COVID-19 (nCVP-118).

**Figure 1:**
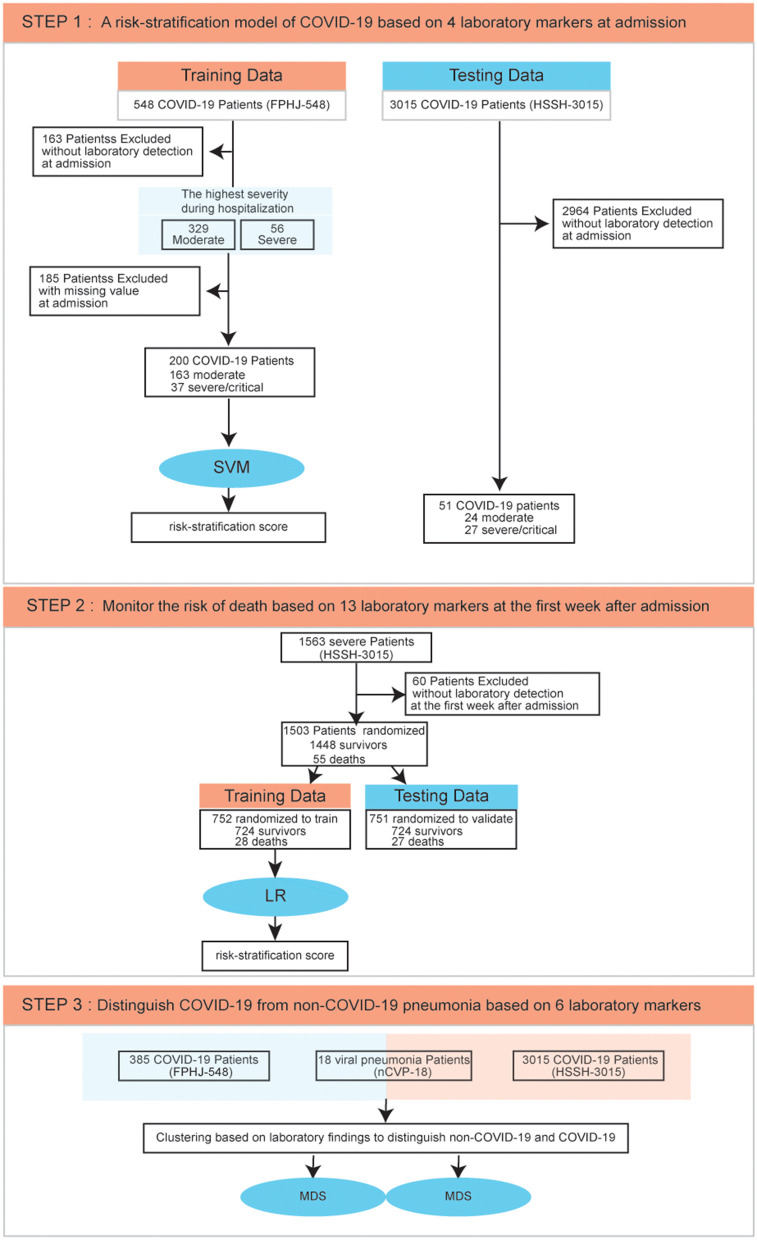
Data process flowchart. SVM, Support Vector Machine; LR, Logistic Regression; MDS, Multidimensional Scaling.

The highest severity during the hospitalization of each patient was recorded, and the laboratory findings of their blood test at admission were used to predict the progression of these patients. In the FPHJ-548 dataset, the average age of these patients was 52.4 (SD: 14.2), and 49.8% were female. Notably, the median age of severe patients was significantly higher than that of moderate patients (Fisher's exact test, *P* < 0.01, Supplementary Table 1). The clinical information of 385 cases (including 329 moderate and 56 severe cases) that had detection data at admission were selected to do the following analysis. To predict the severity of COVID-19 patients at admission, we employed a risk-stratification model based on a support vector machine (SVM) by laboratory indicators in the FPHJ-548 dataset. This model was further validated in an independent dataset (HSSH-3015) (Figure 1, details see Methods).

Then, to monitor the survival outcome of severe COVID-19 patients, we selected 1,448 survival patients and 55 deaths from the HSSH-3015 dataset. 60 patients without laboratory findings within the 1st week since admission were excluded. We randomly split the HSSH-3015 dataset into a leave-in training set and a leave-out test set for data analysis at a ratio of ~1:1. We assembled a logistic regression model (LR) based on laboratory findings in the training set and validated it in the testing set (Figure 1, details see Methods). To distinguish COVID-19 from non-COVID-19 viral pneumonia, we compared the laboratory difference between COVID-19 datasets (FPHJ-548 or HSSH-3015) and the non-COVID-19 dataset (nCVP-118) (Figure 1, details see Methods).

### A Risk-Stratification Model of COVID-19 Based on Four Laboratory Findings at Admission

**Figure 2:**
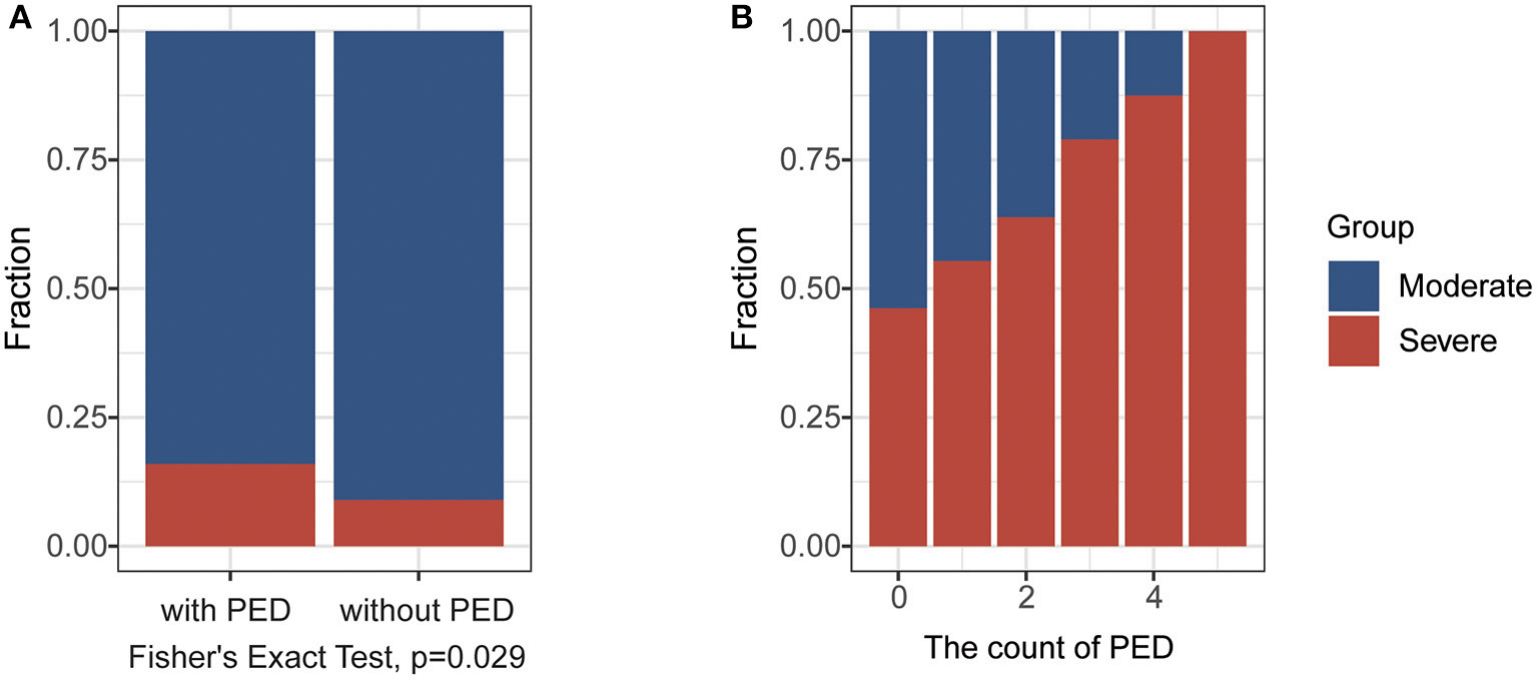
The impact of the pre-existing disease (PED) on the progression of COVID-19. **(A)** The fraction of severe cases in patients with or without in PED in the FPHJ-548 dataset. The red represents the severe COVID-19 patients and the blue represents moderate COVID-19 patients. **(B)** The fraction of severe cases in patients with different numbers of PEDin HSSH-3015 dataset. The red represents the severe COVID-19 patients and the blue represents moderate COVID-19 patients.

According to the highest severity of each patient during hospitalization, we explored the difference in laboratory findings between moderate and severe COVID-19 cases in the FPHJ-548 dataset. We found that high-risk factors related to the progression of COVID-19 included procalcitonin (PCT), C-reactive protein (CRP), neutrophils percentage (NEUT%), lymphocytes percentage (LYMPH%), lactate dehydrogenase (LDH) (Wilcoxon rank-sum test, *P* < 0.001, Table 1). We noted that most of the severe patients presented lymphopenia and elevated levels of inflammatory biomarkers. The levels of PCT in severe patients at the initial stage were higher than those in moderate patients (0.225 vs. 0.06, Wilcoxon rank-sum test, *P* < 0.001), suggesting that serial procalcitonin measurement may play a role in predicting evolution toward a more critical condition (13). The CRP showed a similar trend to PCT, which became significantly higher in severe patients (44.5 vs. 21.8, Wilcoxon rank-sum test, *P* < 0.001). Lymphocyte percentage was significantly higher in the moderate COVID-19 patients than severe COVID-19 patients (22.4 vs. 13.8%, Wilcoxon rank-sum test, *P* < 0.001). The percentage of neutrophils was elevated along with the severity of COVID-19 (77.8 vs. 66.4, Wilcoxon rank-sum test, *P* < 0.001). The LDH (314 vs. 235, Wilcoxon rank-sum test, *P* < 0.001) of severe patients was significantly higher than those of moderate patients. Considering that most of these differential indicators are related to organ damage, we next explored the impact of the pre-existing diseases on the progression of COVID-19. Based on the FPHJ-548 dataset, we found that only 9% of patients without pre-existing disease progressed to severe conditions. In contrast, 16% of severe patients were diagnosed with at least one kind of pre-existing disease (Fisher's Exact Test, *P* = 0.029, Figure 2A), suggesting that COVID-19 patients with a pre-existing disease were prone to develop severe illness. We found the same trend in the HSSH-3015 dataset. Patients with multiple pre-existing diseases were more inclined to progress to severe cases (Figure 2B).

**Table 1:** Comparing laboratory findings between moderate and severe COVID-19 patients.

	**Total** **(*N* = 385)**	**Moderate** **(*N* = 329)**	**Severe** **(*N* = 56)**	***P*-value**
**Laboratory findings**				
**Infection markers**				
Procalcitonin, ng/ml	0.07 (0.04–0.14) (157)	0.06 (0.04–0.11) (130)	0.225 (0.14–0.53) (27)	<0.001
C-reactive protein	24 (12–52) (288)	21.8 (9.4–41) (190)	44.5 (25–120) (38)	<0.001
Lymphocytepercentage, %	21.07 (14–29) (372)	22.4 (16–30) (318)	13.8 (7–21) (54)	<0.001
Monocyte percentage, %	8.98 (6.7–12) (373)	9.6 (7–13) (319)	7.5 (5.4–9.1) (54)	<0.001
Neutrophil percentage, %	67.92 (58–77) (372)	66.4 (57–75) (318)	78.8 (69–86) (54)	<0.001
**Liver injury markers**				
Albumin, g/L	38.95 (36–42) (206)	39.2 (36–43) (167)	37.95 (33–42) (39)	0.039
Creatine Kinase MB	15 (12–19) (185)	14.5 (12–17) (148)	17.85 (18–21) (37)	0.018
Uric acid, umol/L	261 (200–360) (208)	250 (190–330) (169)	337 (270–430) (39)	<0.001
Cholinesterase	6,903 (5,900–8,100) (198)	7,088 (6,000–8,300) (160)	6296.55 (4,600–7,400) (38)	0.007
**Heart injury markers**				
Lactate dehydrogenase, U/L	242.85 (200–330) (215)	235 (200–300) (173)	314 (240–500) (42)	<0.001
N terminal pro B type natriuretic peptide	90.285 (49–410) (81)	80.34 (45–190) (55)	292.1 (78–11,000) (26)	0.0021
High-sensitivity troponin T	0.009 (0.006–0.016) (179)	0.008 (0.006–0.013) (145)	0.016 (0.0095–0.04) (34)	<0.001
**Kidney injury markers**				
Creatinine	66.45 (53–81) (208)	64.85 (51–78) (169)	81.5 (65–330) (39)	<0.001
Glomerular filtration rate	98.7 (80–110) (208)	100.7 (89–110) (169)	85.5 (14–100) (39)	<0.001
Homocysteine	13.75 (11–17) (141)	13 (11–17) (111)	17 (14–27) (30)	<0.001
**Bloodexamination**				
Hematocrit, %	39.85 (36–43) (373)	40.1 (37–43) (319)	37.75 (34–42) (54)	0.002
Hemoglobin, g/L	135.25 (120–150) (373)	136 (120–150) (319)	129.5(110–140) 54)	0.012
Platelet count, 10^9^/L	177 (140–230) (373)	179.5 (150–230) (319)	153 (120–210) (54)	<0.001
Red blood cell count	4.37 (4–4.7) (373)	4.39 (4.1–4.7) (319)	4.23 (3.7–4.6) (54)	<0.001
White blood cell count, g/L	5.7 (4.6–7.4) (372)	5.7 (4.5–7.2) (318)	6.25 (4.9–9.5) (54)	0.01
Platelet volume distribution width	13.4 (12–16) (372)	13.3 (12–16) (318)	15.2 (12–16) (54)	0.023
CO2	21.7 (20–23) (208)	22.1 (20–24) (169)	19.85 (18–21) (39)	<0.001
γ-glutamyltranspeptidase,U/L	25 (17–51) (206)	24 (16–44) (166)	38.5 (24–64) (40)	0.0015
MG	0.9 (0.85–0.96) (204)	0.9 (0.84–0.94) (164)	0.93 (0.89–1) (40)	0.0018
Urea	4.6 (3.5–6) (208)	4.4 (3.4–5.6) (169)	6.45 (4.9–19) (39)	<0.001
Myoglobin	46.64 (24–99) (178)	40.25 (21–81) (144)	106.9 (54–200) (34)	<0.001

The difference in laboratory indicators between severe and moderate patients prompted us to develop a model based on laboratory indicators to predict the state of patients (Figure 1, details see Methods). To validate that whether laboratory findings could predict the progression of COVID-19, we performed t-distributed stochastic neighbor embedding (t-SNE) based on the laboratory indicators in the FPHJ-548 dataset. The result showed that there was an essential difference in laboratory indicators between moderate and severe patients. 95% of the samples were correctly classified (true positive rate:0.66, true negative rate:1, Figure 3A).

**Figure 3:**
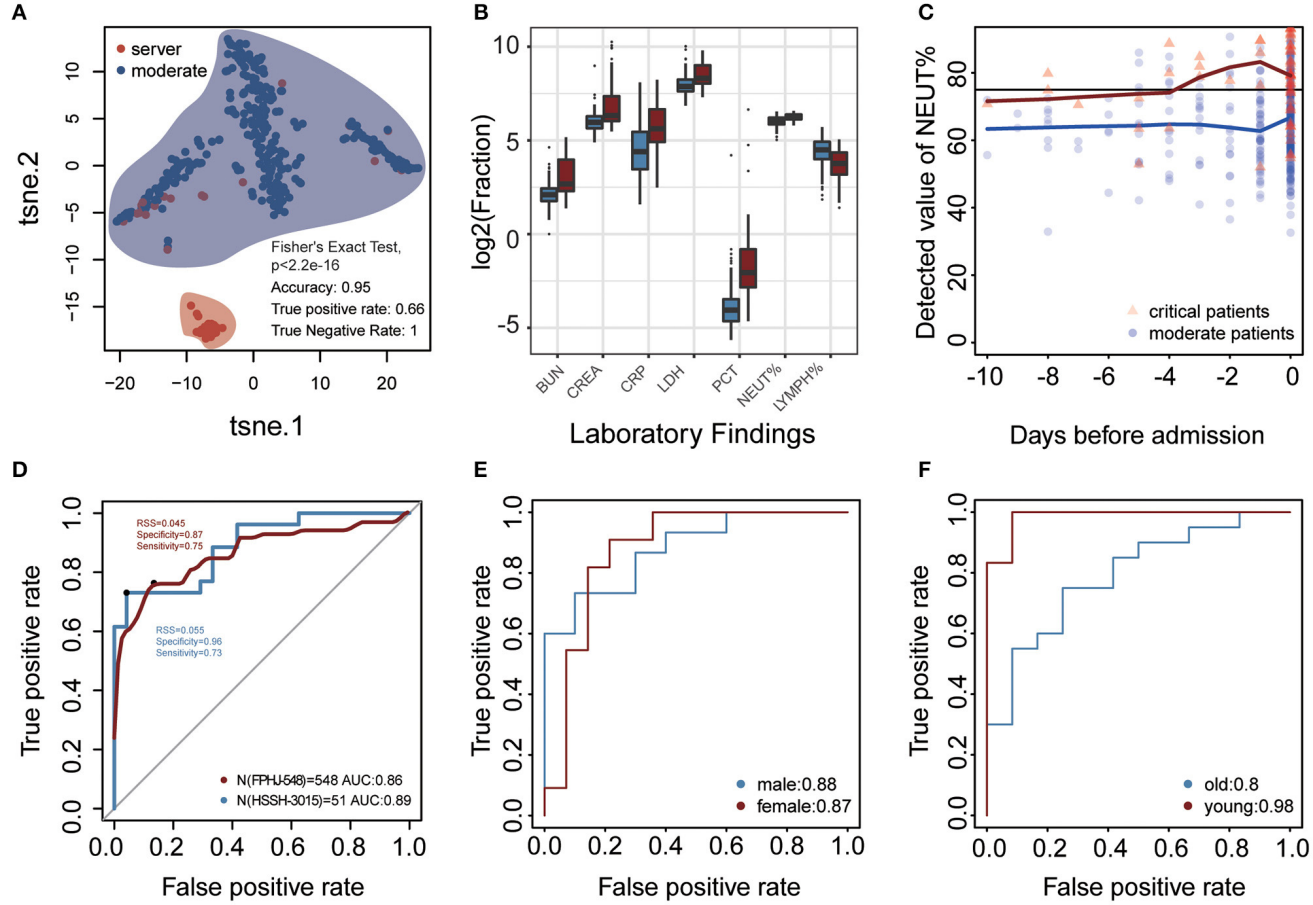
Laboratory findings for predicting progression of COVID-19 at admission. **(A)** t-SNE was performed on 329 moderate COVID-19 patients and 56 severe COVID-19 patients based on 40 laboratory findings (FPHJ-548 dataset). The red represents the severe patients and the blue represents moderate patients. **(B)** The difference of representative clinical markers between moderate and severe patients in the FPHJ-548 dataset. The red represents the severe patients and the blue represents the moderate patients. **(C)** The change of neutrophils percentage during the period before admission. The black line is the maximum reference value. The blue dot represents moderate patients and the red triangle represents severe patients. **(D)** The ability of the model to distinguish severe from moderate patients based on 4 laboratory findings at admission. The x-axis is specificity and the y-axis represents sensitivity. The red solid line represents the mean of the 5-fold cross-validation. The blue represents the AUC of HSSH-3015. **(E,F)** The ability of the model in patients of different sex and age.

For each indicator in FPHJ-548, the correspondent AUC was calculated using the detected value as predictor and the status of progression as an outcome. We selected features whose AUC is >0.7 and only kept indicators that have detection data at admission in both the FPHJ-548 dataset and HSSH-3015 dataset (Supplementary Figure 1). Finally, our model incorporated four indicators, including LYMPH%, NEUT%, creatinine (CREA), and urea nitrogen (BUN) (Figure 3B). The NEUT% between moderate and severe patients showed a noticeable increase at about 4 days before the admission in the FPHJ-548 dataset (Figure 3C). On the contrary, the neutrophil of moderate patients were stable, and between the range of normal reference.

We then applied these four indicators to develop a support vector machine model, followed by 5-fold cross-validations as internal validation. The average sensitivity and specificity of five cross-validations were 0.89 and 0.84, respectively. The average AUC of the five cross-validations was 0.86 (AUC 95% CI:0.84–0.88). The representative receiver operating characteristic (ROC) curve for the external validation (HSSH-3015 dataset) is shown in Figure 3D. It still achieved satisfying results in the testing dataset (sensitivity and specificity, 0.73 and 0.96, respectively, AUC:0.89). Lastly, to avoid the biases of age and sex, we divided patients into two groups by age or sex to test our model. The results showed that our model still had good performance when considering age and sex (Figures 3E,F). To validate the stability of our model at different time points, we considered the status of laboratory indicators within 3, 5, and 7 days after admission. Although the AUC of the model was lower than at admission, our model still had good performance (Mean AUC: 0.71, 0.72, 0.72, respectively for 3, 5, and 7 days after admission, Supplementary Figure 2).

### Laboratory Findings Within the 1st Week After Admission Could Predict the Risk of Death of COVID-19

**Figure 4:**
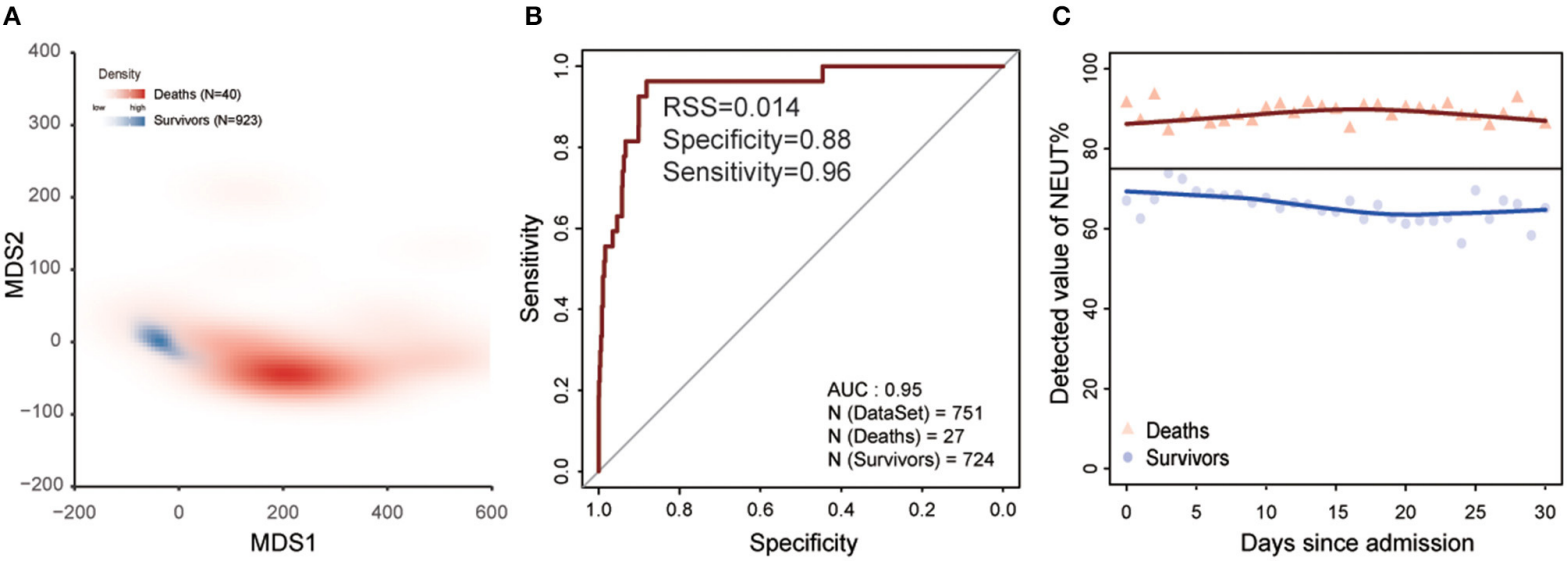
Laboratory findings to predict the clinical outcome of COVID-19 within the 1st week after admission. **(A)** MDS plot for distinguishing deaths from survivors based on 13 laboratory findings in training dataset. Red represents deaths and blue represents survivors. **(B)** The ability of the model for distinguishing deaths from survivors based on 13 laboratory findings in the test dataset. The x-axis is specificity and the y-axis represents sensitivity. **(C)** The change in the percentage of neutrophils since admission. The black line is the maximum reference value. The blue dot represents survivors and red represents deaths.

The progression of COVID-19 into severe illness increases the risk of death, so we predicted the survival outcome of severe patients in the HSSH-3015 dataset based on the laboratory findings within the 1st week after admission (Figure 1). Patients were randomly divided into a training group and validation group at the ratio of 1:1. To avoid the deviation caused by the difference between the number of deaths and the number of survivors, we randomly selected the surviving patients so that the number of surviving patients equals the number of dead patients. We used stepwise logistic regression to identify the important laboratory indicators. This process was repeated 100 times (details see Methods). Thirteen indicators with statistically significant differences between survivors and deaths were identified. These were Albumin/Globulin, DD dimer, leukocyte, monocytes, cystatin C, creatinine, lymphocyte, urea nitrogen, thrombin time, prothrombin time, lactate dehydrogenase, fibrinogen, percentage of neutrophils. We performed multidimensional scaling in the training dataset based on these 13 markers. Results show that these indicators could distinguish deaths from survival (accuracy = 0.96, true positive rate: 0.82, true negative rate: 0.97, Figure 4A). Then, based on these 13 indicators, we developed a logistic model to predict the survival outcome in the training dataset. We found that the model predicts the survival outcome with high accuracy in the testing dataset (AUC = 0.95, Figure 4B). The average NEUT% of dead patients exceeded the maximum normal value during hospitalization. In contrast, the neutrophil of survivors was stable, and between the range of normal reference (Figure 4C).

### Distinguishing COVID-19 From Non-COVID-19 Viral Pneumonia Based on Laboratory Findings

**Figure 5:**
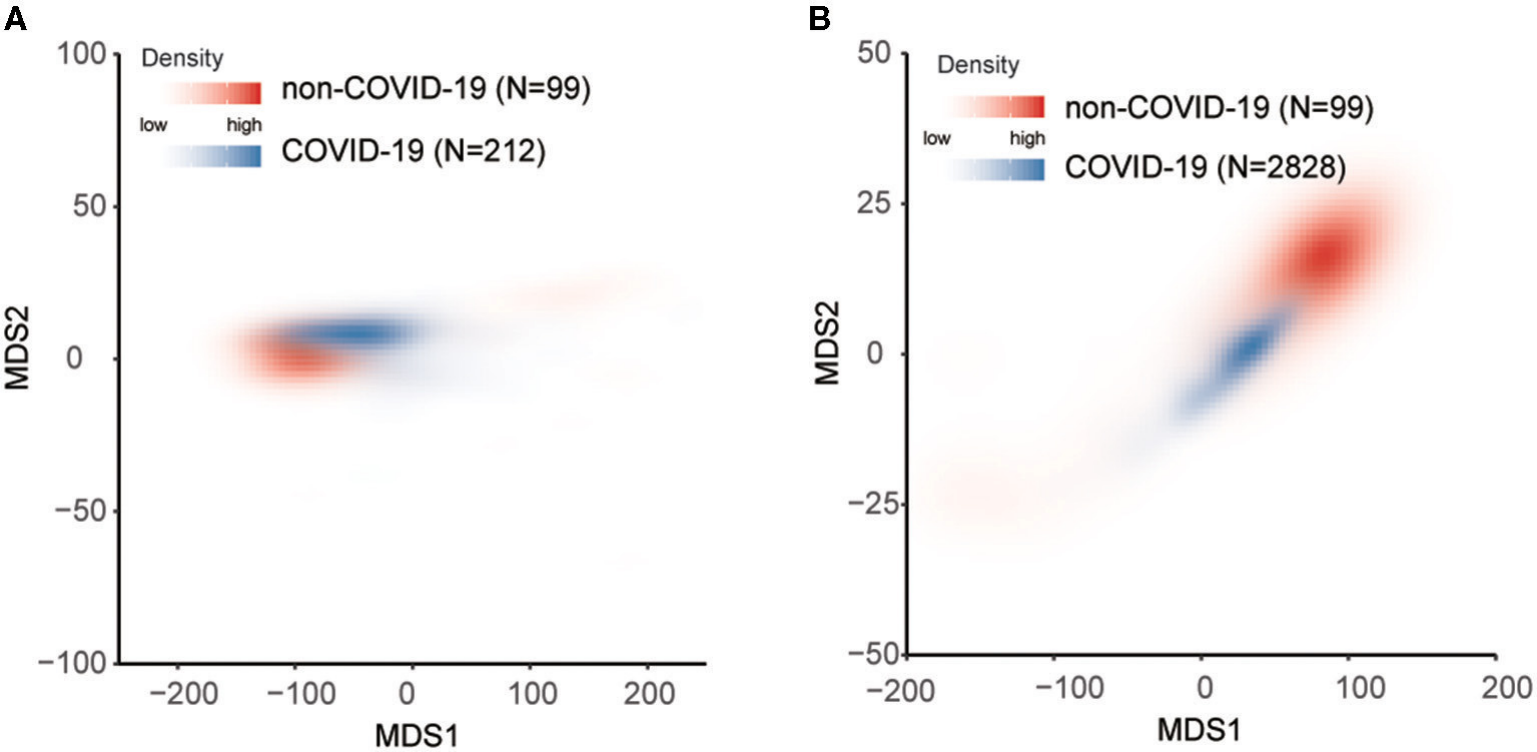
MDS plot for distinguishing non-COVID-19 from COVID-19 based on laboratory findings. Red represents non-COVID-19 and blue represents COVID-19. The depth of the color represents the density. **(A)** The difference between FPHJ-548 and nCVP-118 dataset. **(B)** The difference between nCVP-118 and the HSSH-3015 dataset.

Increasing studies have shown that the infection of viral pneumonia might be associated with organ dysfunction (14–17). Hence, we explored the change of organ function-related indicators between FPHJ-548 and nCVP-118. Interestingly, we found that some indicators related to organ dysfunction showing significant differences between the two groups (Table 2, Wilcoxon two-sided rank-sum test, *P* < 0.05). Our studies showed that patients in the non-COVID-19 group had higher levels of WBC than those of the COVID-19 group (6.65L vs. 5.7, *P* = 0.002). Besides, the level of LDH in the non-COVID-19 group was lower than that of COVID-19 patients (182 vs. 242.85, *P* < 0.001). The level of aspartate transaminase (22 vs. 27.4, *P* < 0.001) was higher in the COVID-19 group. To further confirm the reliability and stability of the above results, we randomly selected 20 COVID-19 patients and 20 non-COVID-19 patients to compare the difference in laboratory indicators. This process was repeated 100 times. The different indicators are consistent across 100 iterations (Supplementary Figure 3). We calculated the permutation *p*-value for each indicator to identify the significant indicators in most of the random sampling. Six indicators with significant differences permutation *p* values were selected (permutation *p* < 0.05), including eosinophil percentage, white blood cell count, albumin, aspartate transaminase, lactate dehydrogenase, and hemoglobin. Hence, we used these laboratory findings to perform multidimensional scaling among FPHJ-548, nCVP-118, HSSH-3015, and nCVP-118 (Figure 5A, details see Methods). We found that these indicators can distinguish COVID-19 and non-COVID-19. For verification, we performed the same method on HSSH-3015 and nCVP-118 and found similar results (Figure 5B).

**Table 2:** Comparing laboratory findings between COVID-19 and non-COVID-19 patients.

	**Normal range**	**Total** **(*N* = 403)**	**Non-COVID-19** **(*N* = 118)**	**COVID-19** **(*N* = 385)**	***P*-value**	**Adjust** ***P*-value**
**Laboratory findings**						
**Infection markers**						
Eosinophil percentage, %	0.4~8	0.64 (0.2–1.3)	1.1 (0.5–1.9)	0.5 (0.2–1.2)	<0.001	0.007
White blood cell count, g/L	3.5~9.5	5.9 (4.6–7.6)	6.65 (5.1–8.3)	5.7 (4.6–7.4)	0.002	0.045
**Liver injury markers**						
Albumin, g/L	35~55	38.4 (35–42)	36.8 (33–41)	38.95 (36–42)	<0.001	0.041
Alanine transaminase	9~50	18.2 (12–29)	16.5 (11–23)	19 (12–29)	0.1	0.272
Aspartate Transaminase	13~35	25.8 (19–36)	22 (17–28)	27.4 (20–41)	<0.001	0.043
**Heart injury markers**						
Lactate Dehydrogenase, U/L	80~285	231 (180–320)	182 (150–300)	242.85 (200–330)	<0.001	0.002
N-terminal pro-brain natriuretic peptide	0~125	76.8 (49–240)	65.25 (48–120)	90.285 (49–410)	0.067	0.086
**Blood examination**						
Activated partial thromboplastin time	24~36	30.3 (28–32)	31 (29–36)	30.1 (28–32)	0.0039	0.16
Hematocrit, %	40~50	39.6 (36–43)	38.4 (34–41)	39.85 (36–43)	0.001	0.113
Hemoglobin, g/L	130~175	134 (120–150)	127.5 (110–140)	135.25 (120–150)	<0.001	0.031
Calcium, mmol/L	2.08~2.8	2.12 (2–2.2)	2.2 (2.1–2.3)	2.1 (2–2.2)	<0.001	0.075
Potassium, mmol/L	3.5~5.3	4 (3.7–4.3)	3.9 (3.7–4.3)	4.02 (3.7–4.4)	0.25	0.299
Sodium, mmol/L	137~147	139 (136–141)	139 (138–140)	139 (136–141)	0.46	0.474

Considering that these indicators were related to liver disease and heart disease, we removed the patients with liver and heart disease in the HSSH-3015 dataset to exclude the impact of pre-existing disease. Results showed that these indicators could still differentiate COVID-19 from non-COVID-19 (Supplementary Figure 4). In summary, these findings demonstrated that laboratory findings can distinguish COVID-19 patients from non-COVID-19 patients.

## Discussion

About 6.5% of COVID-19 patients experience a sudden progression to severe conditions, with a fatality rate of 49% in these patients (18). There is an urgent need for effective methods of predicting and monitoring the progression of COVID-19 patients from moderate to severe conditions. First, based on the FPHJ-548 dataset, we systematically explored the difference in laboratory findings between severe and moderate patients. We found that the high-risk factors related to the progression of COVID-19 included PCT, CRP, NT-proBNP, neutrophils percentage, LDH, and LYMPH%, etc. Most of these laboratory indicators were reported to be associated with the progression of COVID-19 (19). The lymphocyte count was lower in non-survivors than survivors (11). Severe cases presented lower lymphocyte counts and higher neutrophil levels (11, 20). LDH was found to be a risk factor associated with disease progression in patients infected with COVID-19 (21). Many types of research have proven that elevated NT-proBNP was significantly correlated with critical disease (22). Initial blood urea nitrogen and serum creatinine were related to increased mortality in COVID-19 (23, 24). In addition, we found that the proportion of severe conditions was positively associated with the increase in the number of pre-existing diseases diagnosed in the patients. Many studies demonstrated that these pre-existing diseases might promote the expression of ACE2 (25, 26), leading to a high-risk of COVID-19 infection. Based on a set of laboratory indicators (NEUT%, LYMPH%, CREA, and BUN), we finally constructed a risk-stratification model by using an SVM model, achieved the AUC of 0.89 in an independent dataset. Then, we based it on 13 laboratory findings ensemble a model to predict survival outcome with high accuracy. At last, we proved that laboratory findings could distinguish COVID-19 patients from non-COVID-19 patients. In the latest research, Zhang et.al. have developed an artificial intelligence (AI) tool, which could classify the severity and predict critical illness based on chest CT images and laboratory indicators (7). The deep learning survival Cox model was also developed to predict the clinical outcome of COVID-19 patients with high accuracy. This model uses ten clinical variables, including common demographic and clinical characteristics, as well as laboratory results (27). However, in the emergency of the pandemic, the requirements of professional devices and clinicians make these methods difficult to use rapidly. Our model used four laboratory indicators that are available at most hospitals and achieve comparable sensitivity and specificity. When the medical system is overloaded in a pandemic or rural area, this risk-stratification model can help screen patients who may develop severe illness accurately by easy detection and low-cost testing, as early as admission.

Our model has some limitations. First, because of the emergency of the epidemic, some patients did not take the blood tests at admission, which limited the power of prediction and validation. Second, further studies on different populations with larger patient cohorts are required to verify our findings, especially in distinguishing COVID-19 from non-COVID-19 viral pneumonia based on laboratory findings. As the tendency of organ dysfunction of COVID-19 and other pneumonia is controversial currently, more extensive comparison analysis is needed to validate the difference (28, 29). Our practical prognostic model, based on laboratory indicators, is a convenient and effective method of risk-stratification for COVID-19 at admission, providing a way of ensuring that severe patients receive treatment early, and enabling medical resources to be allocated effectively. Our study provides vital information for clinical practice in the diagnosis and monitoring of COVID-19 patients.

## Data Availability Statement

The original contributions presented in the study are included in the article/Supplementary Material, further inquiries can be directed to the corresponding author/s.

## Ethics Statement

The studies involving human participants were reviewed and approved by the Medical Ethical Committee of the First People's Hospital of Jiangxia District of Wuhan (2020029), Wuhan Huoshenshan Hospital (HSSLL011). The protocol of this study was approved by the Ethical Committee of Nanjing Medical University (2020-511). The patients/participants provided their written informed consent to participate in this study.

## Author Contributions

SW, PL, XX, and KL had full access to all of the data in the study and all take responsibility for the integrity of the data analysis, and contributed to concept and design. CL, CX, WH, HS, YW, ZN, YG, and RZ undertook data collection. ZW, WW, LW, JL, QW, and HT undertook data analysis and interpretation. All authors contributed to the article and approved the submitted version.

## Conflict of Interest

The authors declare that the research was conducted in the absence of any commercial or financial relationships that could be construed as a potential conflict of interest.

## Publisher's Note

All claims expressed in this article are solely those of the authors and do not necessarily represent those of their affiliated organizations, or those of the publisher, the editors and the reviewers. Any product that may be evaluated in this article, or claim that may be made by its manufacturer, is not guaranteed or endorsed by the publisher.

## Supplementary Material

Supplementary Figure 1 The receiver operating characteristic (ROC) curve of identified indicators to distinguish severe and moderate patients in the FPHJ-dataset.

Supplementary Figure 2 The receiver operating characteristic (ROC) curve of risk-stratification score to distinguish severe and moderate patients within 3, 5, 7 days after admission in the HSSH-3015 dataset. **(A)** The model performance within 3 days. **(B)** The model performance within 5 days. **(C)** The model performance within 7 days.

Supplementary Figure 3 The difference of laboratory findings between non-COVID-19 and COVID-19. 20 patients were randomly selected without replacement from the nCVP-118 dataset and FPHJ-548 dataset, respectively. This process was repeated 100 times. The y-axis represents the median value in each iteration. ^**^*P* < 0.01.

Supplementary Figure 4 MDS plot for distinguishing non-COVID-19 from COVID-19 without liver or heart disease based on laboratory findings. Red represents non-COVID-19 and blue represents COVID-19. The depth of the color represents the density.

Supplementary Table 1 Clinical characteristics of patients in the FPHJ-458 dataset.

## Abbreviations

COVID-19: the coronavirus disease 2019
ALP: Alkaline phosphatase
FPHJ-548: data of 548 patients from First People's Hospital of Jiangxia District of Wuhan
HSSH-3015: data of 3015 patients from Wuhan Huoshenshan Hospital
nCVP: non-COVID-19 viral pneumonia
SVM: support vector machine
LR: logistic regression
PCT: procalcitonin
CRP: C-reactive protein
NEUT%: neutrophils percentage
LYMPH%: lymphocytes percentage
LDH: lactate dehydrogenase
t-SNE: t-distributed stochastic neighbor embedding
CREA: creatinine
BUN: urea nitrogen
ROC: receiver operating characteristic
AI: artificial intelligence
RSS: risk-stratification score
PED: pre-existing disease.
