# Supplementary figures and images for: Laboratory Testing Implications of Risk-Stratification and Management of COVID-19 Patients
Source: Front Med (Lausanne). 2021 Aug 13;8:699706. doi: 10.3389/fmed.2021.699706 (PMC8414546; doi:10.3389/fmed.2021.699706)

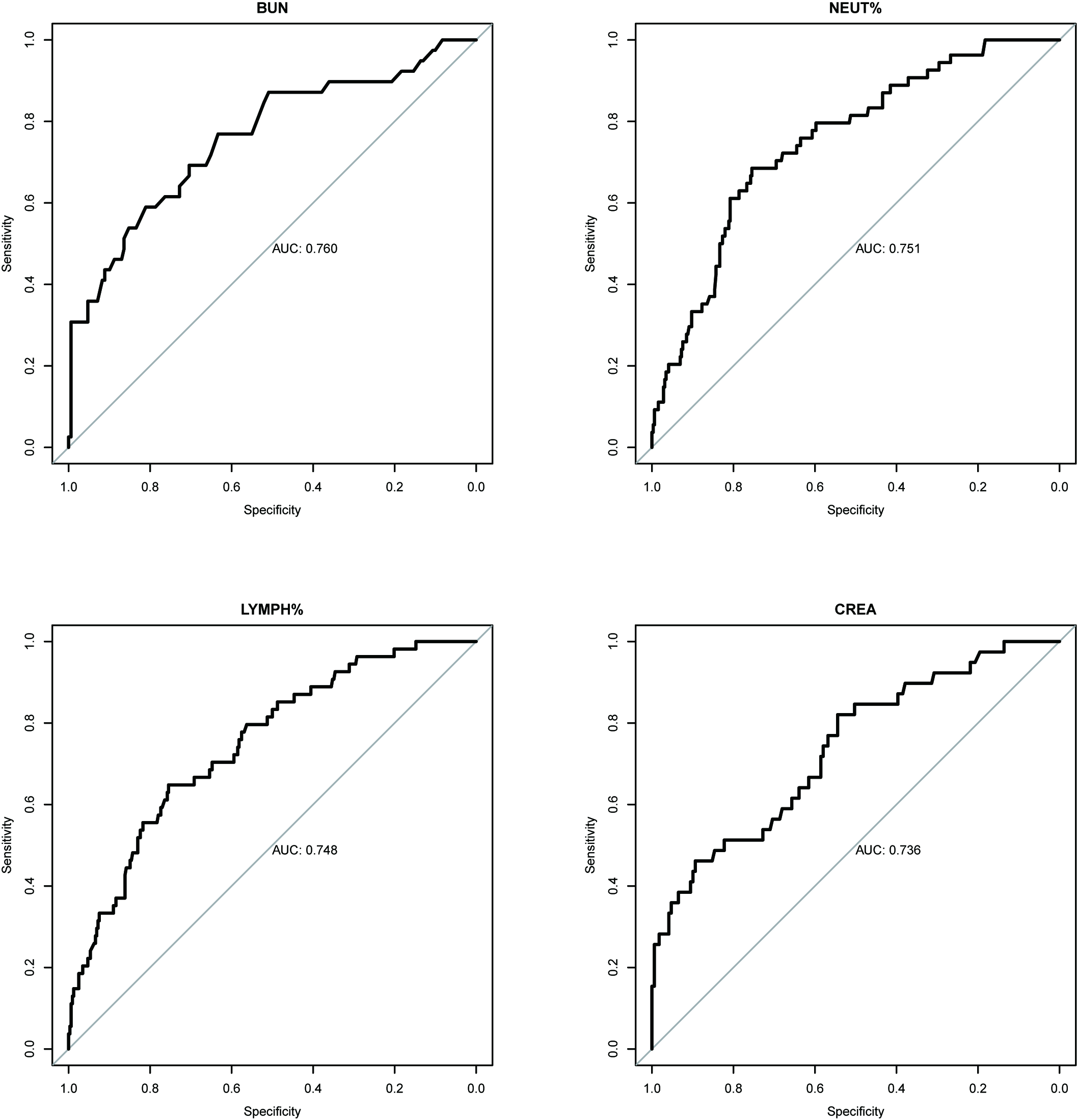

Supplement: Supplementary Figure 1 — The receiver operating characteristic (ROC) curve of identified indicators to distinguish severe and moderate patients in the FPHJ-dataset. [file Image_1.TIF]

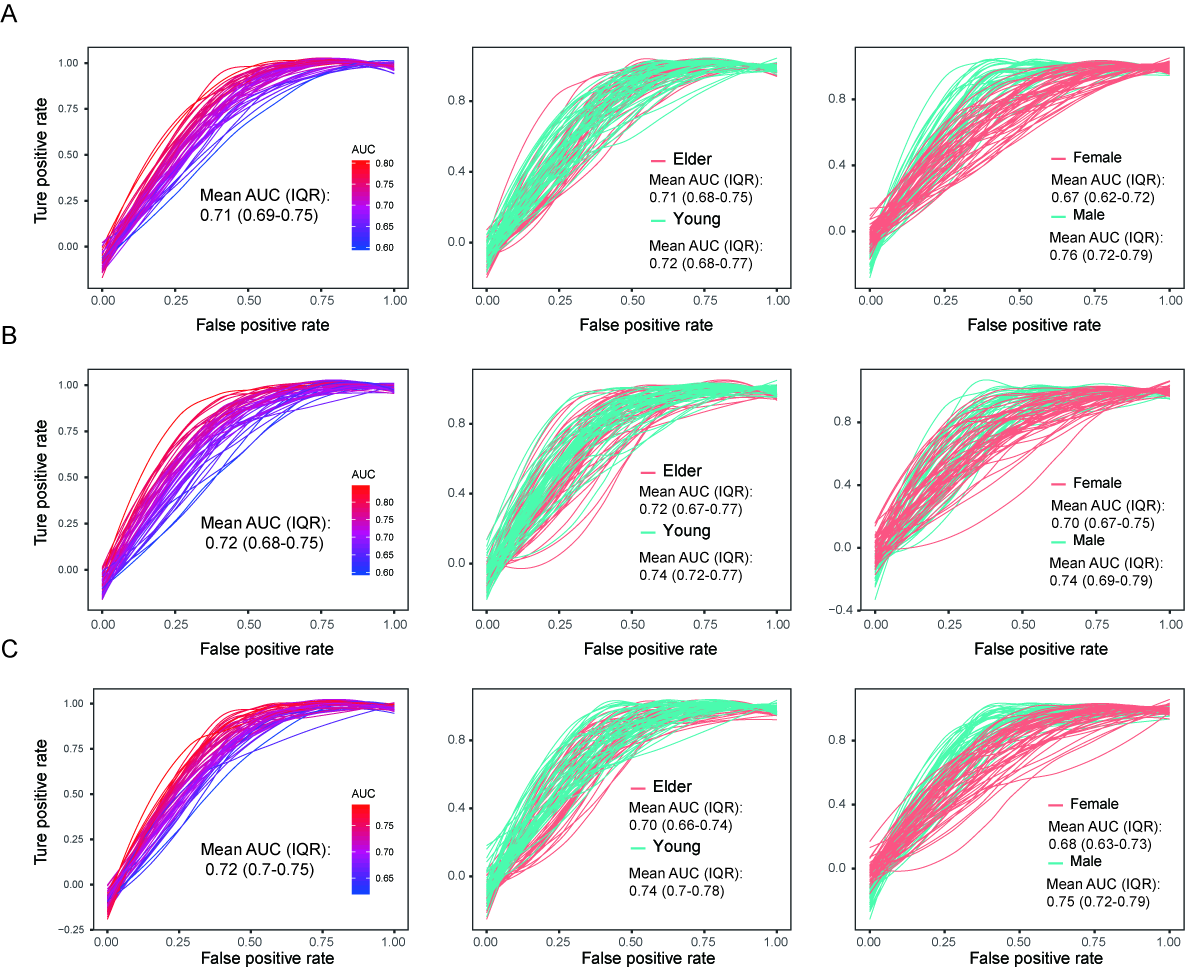

Supplement: Supplementary Figure 2 — The receiver operating characteristic (ROC) curve of risk-stratification score to distinguish severe and moderate patients within 3, 5, 7 days after admission in the HSSH-3015 dataset. (A) The model performance within 3 days. (B) The model performance within 5 days. (C) The model performance within 7 days. [file Image_2.TIF]

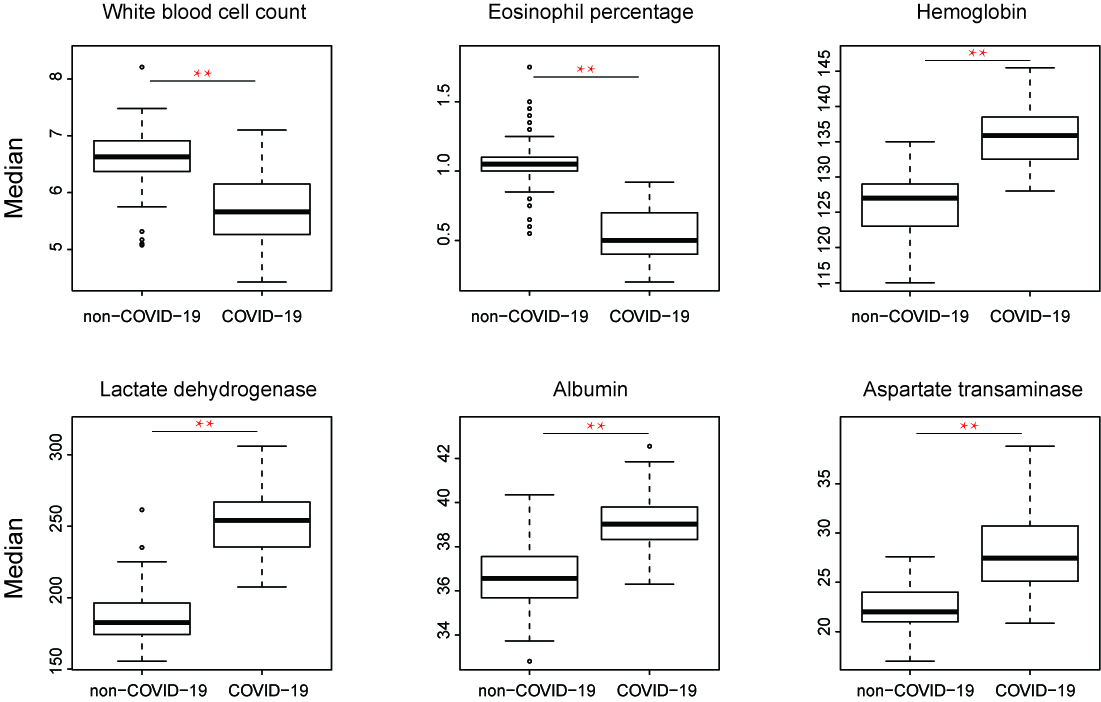

Supplement: Supplementary Figure 3 — The difference of laboratory findings between non-COVID-19 and COVID-19. 20 patients were randomly selected without replacement from the nCVP-118 dataset and FPHJ-548 dataset, respectively. This process was repeated 100 times. The y-axis represents the median value in each iteration. **P < 0.01. [file Image_3.TIF]

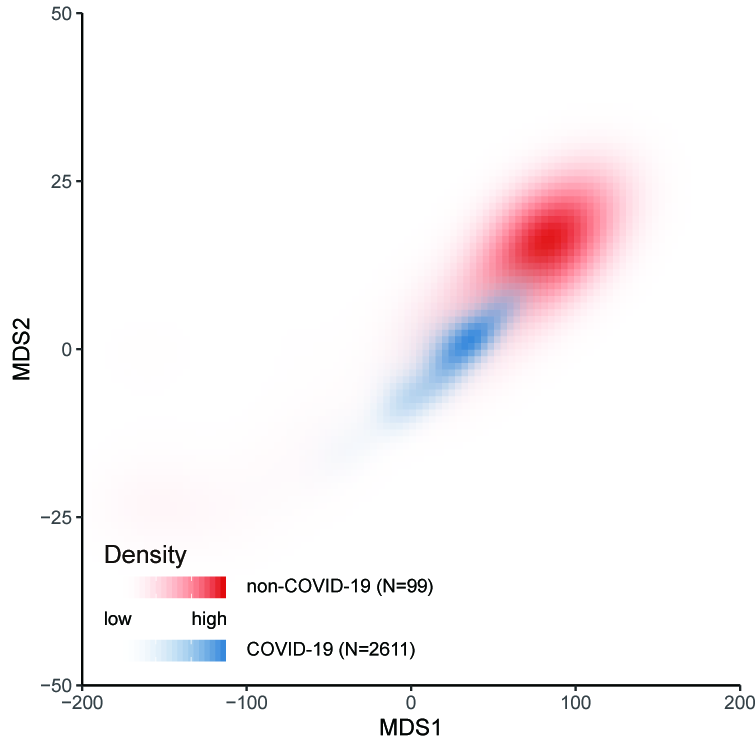

Supplement: Supplementary Figure 4 — MDS plot for distinguishing non-COVID-19 from COVID-19 without liver or heart disease based on laboratory findings. Red represents non-COVID-19 and blue represents COVID-19. The depth of the color represents the density. [file Image_4.TIF]
