# Supplementary material for: Laboratory Testing Implications of Risk-Stratification and Management of COVID-19 Patients
Source: Front Med (Lausanne). 2021 Aug 13;8:699706. doi: 10.3389/fmed.2021.699706 (PMC8414546; doi:10.3389/fmed.2021.699706)
Supplement: Supplementary Table 1 — Clinical characteristics of patients in the FPHJ-458 dataset. [file Table_1.docx]

**Supplementary Table 1.** Clinical characteristics of patients in the FPHJ-458 dataset.

|  | Total  (n=205^*^) | Moderate  (n=148) | Severe  (n=57) | P value |
| --- | --- | --- | --- | --- |
| Age (x±s) | 52.4±14.2 | 49.9±13.2 | 59.1±14.3 | <0.01 |
| Gender |  |  |  |  |
| Male(n,%) | 103 (50.2) | 75 (50.7) | 28 (49.1) | 0.84 |
| Female(n,%) | 102 (49.8) | 73 (49.3) | 29 (50.9) | 0.84 |
| Wuhan residence history or close contact with confirmed cases(n,%) | 69 (33.7) | 51 (34.5) | 18 (31.6) | 0.70 |
| Comorbidities |  |  |  |  |
| COPD | 2 (1.0) | 1 (0.7) | 1 (1.8) | 0.48 |
| Diabetes(n,%) | 15 (7.3) | 6 (4.1) | 9 (15.8) | 0.004 |
| Hypertension(n,%) | 22 (10.7) | 7 (4.7) | 15 (26.3) | <0.001 |
| Chronic liver disease(n,%) | 10 (4.9) | 6 (4.1) | 4 (7.0) | 0.37 |
| Chronic kidney disease(n,%) | 9 (4.4) | 4 (2.7) | 5 (8.8) | 0.057 |
| Cerebrovascular disease(n,%) | 12 (5.9) | 1 (0.7) | 11 (19.3) | <0.001 |
| Cardiovascular disease(n,%) | 11 (5.4) | 4 (2.7) | 7 (12.3) | 0.006 |
| Malignancy(n, %) | 3 (1.5) | 1 (0.7) | 2 (3.5) | 0.13 |
| Symptoms |  |  |  |  |
| Dry cough(n,%) | 162 (79.0) | 117 (79.1) | 45 (78.9) | 0.99 |
| Diarrhea(n,%) | 13 (6.3) | 8 (5.4) | 5 (8.8) | 0.38 |
| Fever(n,%) | 123 (60.0) | 85 (57.4) | 38 (66.7) | 0.23 |
| Dyspnea(n,%) | 14 (6.8) | 9 (6.1) | 5 (8.8) | 0.49 |
| Expectoration | 25 (12.2) | 16 (10.8) | 9 (15.8) | 0.33 |
| Myalgia | 61 (29.8) | 47 (31.8) | 15 (26.3) | 0.45 |
| Nausea | 18 (8.8) | 12 (8.1) | 6 (10.5) | 0.58 |
| Headache | 12 (5.9) | 9 (6.1) | 3 (5.3) | 0.82 |
| Vomiting | 8 (3.9) | 4 (2.7) | 4 (7.0) | 0.15 |
| Fatigue | 102 (49.8) | 73 (49.3) | 29 (50.9) | 0.84 |
| Signs |  |  |  |  |
| Pluse,median(IQR) | 86 (79~93) | 87 (81~93) | 84 (75~94) | 0.12 |
| Systolic pressure(x±s),mmHg | 125.6±11.3 | 122.3±9.7 | 129.7±12.4 | <0.01 |
| Diastolic pressure (x±s),mmHg | 77.9±10.2 | 75.6±9.4 | 81.5±10.9 | <0.01 |
| Respiratory rate,median (IQR) | 20 (19~21) | 20 (19~22) | 20 (19~20) | 0.35 |

COPD: Chronic obstructive pulmonary disease; IQR: interquartile range.

^*^205 patients out of 548 people was with the information of demographics.
